# Review Articles on Soccer Performance Analysis: A Bibliometric Analysis of Current Trends and Emerging Themes

**DOI:** 10.3390/sports13050131

**Published:** 2025-04-24

**Authors:** Spyridon Plakias

**Affiliations:** Department of Physical Education and Sport Science, University of Thessaly, Karyes, 42100 Trikala, Greece; spyros_plakias@yahoo.gr

**Keywords:** association football, science mapping, tactics, physical condition, sports medicine

## Abstract

Objectives: This study aimed to analyze review articles on soccer performance analysis (PA) using a bibliometric approach to address three main research questions: (a) How has the publication of review articles related to soccer PA evolved over time? (b) Which authors and journals have been the most influential in this field and what are the collaboration networks between them? (c) What are the dominant topics, methodological issues, and gaps in the relevant literature? Methods: A systematic search was conducted in the Scopus database on 24 January 2025, using a comprehensive Boolean expression to identify relevant review articles. The extracted data were analyzed using VOSviewer (1.6.20.0). We employed bibliometric performance analysis, science mapping, and clustering techniques including co-authorship, co-occurrence, bibliographic coupling, and co-citation analyses. Results: The study included 314 review articles, demonstrating an exponential increase in publications since 2017. The most influential journal was *Sports Medicine*, while the leading authors included F. M. Clemente and H. Sarmento. Co-occurrence analysis revealed five thematic clusters covering physical performance, nutrition, coaching strategies, tactical analysis, and emerging data-driven approaches. Notably, significant gaps in the literature were identified in the areas of set pieces, key performance indicators, and contextual variables. Conclusions: This bibliometric analysis, creating a comprehensive map of the review articles on soccer PA, highlights the diverse and interdisciplinary nature of soccer PA research, identifies gaps in the literature, and offers practical considerations for researchers and journal editors aiming to advance the field of soccer PA.

## 1. Introduction

Soccer, the world’s most popular sport, significantly impacts everyday life, shaping economies, social dynamics, and national identities [1,2]. This influence has made soccer a subject of extensive research, with approximately 60% more publications than the next most studied sport. Among the various topics, performance analysis (PA) dominates the field, especially since the advent of semi-automated systems have facilitated notational analysis [3].

In any field where there are many studies, it is a challenge for researchers to synthesize relevant findings by conducting review studies. Reviews do not introduce new data but rather synthesize, interpret, and evaluate existing published or presented information [4]. There are various types of review articles, some of which follow stricter methodologies (e.g., systematic, scoping, pyramid reviews) [5,6], while others employ more flexible approaches (e.g., narrative, integrative, and critical reviews) [7,8]. Additionally, there are reviews that utilize qualitative research methodologies (e.g., thematic analysis and grounded theory) [9,10], whereas meta-analyses statistically aggregate the findings of quantitative studies to provide a more accurate estimation of the effects [11].

Sports PA is the assessment of athletes’ performance using different methods for different purposes and is distinguished into theoretical (TPA) and practical (PPA) [12]. TPA is the study of sports performance structures, aiming to identify general laws, quantify the determinants of competition outcomes, and develop models. In contrast, PPA involves analyzing performance to identify strengths, weaknesses, and strategies, ultimately providing actionable recommendations to support the goals of teams or athletes in practice [12]. PA in soccer can provide valuable information regarding tactics, techniques, physical demands, talent detection and development, and injury prevention [13,14,15].

The last attempt to review the broad spectrum of PA in soccer was carried out in 2013 [16], and has even been the subject of debate among scientists [17]. Since then, there has been a sharp increase in research on soccer in general [3], particularly on soccer PA. This growth can be attributed not only to the variety of technical–tactical variables generously provided by data providers such as OPTA, Instatscout, and Wyscout [18] but also to FIFA’s decision in 2015 to allow the use of Electronic Performance and Tracking Systems (EPTS) in soccer teams [19]. It is worth noting that the use of EPTS was allowed not only in elite men’s competitions, but also in women’s and developmental soccer [20,21,22,23]. EPTS offer vast amounts of tracking data, leading to the era of big data and unlocking its potential to support soccer PA [24]. These developments have led to a significant rise in publications on soccer PA, making it impractical to conduct comprehensive reviews using systematic methods such as systematic or scoping reviews across the entire spectrum of soccer PA. Instead, researchers have focused on systematic reviews of more specific topics within soccer PA, such as the effect of Video Assistant Referee (VAR) on match performance [25]; negative effects of mental fatigue on performance [26]; elite-level defensive performance [27]; teams’ and players’ playing styles [13,28]; injury prevention using acute to chronic workload ratio [29]; and velocity and accelerometer thresholds in soccer [30]. In contrast, bibliometric analysis offers a holistic, quantitative mapping of large research landscapes, making it particularly valuable when the aim is to explore broad trends, influential authors and journals, collaboration networks, and thematic clusters—dimensions not typically captured by narrative or systematic reviews.

In the academic literature, bibliometric analysis has proven to be a powerful tool for addressing such challenges. Bibliometric techniques have become widely used and comprehensive methods for analyzing and evaluating large volumes of scientific data [31]. Researchers have applied these techniques for various purposes, including identifying emerging trends in articles and journal performance, understanding collaboration networks and research components, and investigating the intellectual framework of a specific field within the existing literature [32]. 

In recent years, bibliometric analyses have been employed to explore various aspects of soccer. For instance, studies have investigated research trends in youth soccer [33], women’s soccer [34], soccer biomechanics [35], neuropsychophysiological aspects of soccer performance [36], and the impact of heat exposure on soccer players’ health and performance [37]. Regarding soccer PA, Verma and Shrivastava [38] examined research trends in soccer analytics from 2012 to 2023. However, despite its utility and application in soccer-related studies, bibliometric analysis has not yet been applied to provide a comprehensive synthesis of available data across review articles on soccer PA, leaving a significant gap in understanding the evolution and current state of this research field.

The present study seeks not only to fill this gap, but also to address the challenge of the large volume of scientific data in the field of soccer PA, utilizing the Scopus database as the data source. Scopus, renowned for its comprehensive coverage of peer-reviewed scientific publications, offers a reliable and wide platform for inclusive content coverage [39]. This study focused on answering three main research questions: (1) How has the writing of review articles related to soccer PA evolved over time? (2) Which authors and journals have been the most influential in this field and what are the collaboration networks between them? (3) What are the dominant topics, methodological issues, and gaps in the relevant literature?

Ultimately, this study aims to create a comprehensive map of review articles on soccer PA, providing a valuable resource for both current researchers and those entering the field. By systematically analyzing publication trends, identifying leading contributors, and exploring thematic developments, methodological issues, and research gaps, this study may offer a basis for advancing knowledge in soccer PA and underscoring the field’s critical role in shaping the future of soccer.

## 2. Material and Methods

### 2.1. Search

The search for relevant articles was conducted on 24 January 2025, in the Scopus database within the title, abstract, and keyword fields. No temporal or geographical filters were applied during the search process, in order to ensure the inclusion of all relevant review articles regardless of their publication year or country of origin. The Boolean expression consisted of three parts, which were connected using the AND operator. In the first part, terms commonly used in sports PA were included with the OR operator linking them. The second part was used to specify the sport (soccer or football), and the third part was utilized to narrow down the articles to review articles only.

As a result, the final Boolean expression was: (TITLE-ABS-KEY (“performance analysis” OR “notation analysis” OR “notational analysis” OR “match analysis” OR “game analysis” OR “video analysis” OR “motion analysis” OR “fitness” OR “running performance” OR “physical performance” OR “training load” OR “match performance” OR “running variables” OR “technical-tactical variables” OR “contextual variables” OR “situational variables” OR “playing styles” OR “game model” OR “performance indicators” OR “performance profile” OR “performance profiling” OR “analytics” OR “physiological demands” OR “tactics” OR “total distance” OR “sprints” OR “goal scoring”) AND TITLE-ABS-KEY (soccer OR football) AND TITLE-ABS-KEY (review)).

### 2.2. Selection Criteria

Articles obtained from the initial search were screened for eligibility based on specific criteria. Specifically, our study included only review articles, written in English and published in academic journals, with the primary focus of the article being PA in soccer. The articles needed to be concerned with active soccer players of any sex, level or age, but not recreational athletes. Additionally, articles that did not focus exclusively on soccer, but included other sports, were excluded. In this review, the term “performance analysis” is used in a broad and inclusive manner, encompassing not only technical and tactical aspects, but also physical, physiological, and psychological parameters that influence individual and team performance in soccer. Table 1 presents the inclusion–exclusion criteria in detail. The inclusion of an article in the study was primarily determined based on the title and abstract; however, whenever necessary, the full article was read, provided the author had access to it. Although the Boolean expression included the term “review” in the title, abstract, or keywords, each article was individually screened by the author to confirm that it met the criteria of a review article. We did not rely solely on the Scopus classification, as automated categorizations are occasionally inaccurate. Formal frameworks such as PRISMA or AMSTAR were not applied, as the present study is a bibliometric analysis rather than a systematic review. If an article was not available via open access, we assessed its eligibility based on the information provided in the title and abstract. If these were sufficient to determine that the article met all inclusion criteria, it was included in the study. Otherwise, if eligibility could not be confirmed and the full text was not accessible, the article was excluded.

### 2.3. Data Extraction and Software

Information for the articles included in the study was extracted from Scopus in the form of a CSV file. This file contained all available information provided by the database for each article, except for the funding details. The CSV file was then imported into the open-source software VOSviewer (1.6.20.0) to conduct the bibliometric analysis, and additional charts required for the study were created using Microsoft Power BI (Office 365, Microsoft Corporation, Redmond, WA, USA). Furthermore, draw.io, an open-source web-based application, was used to create the flow diagram [40,41].

### 2.4. Bibliometric Analysis

To conduct this bibliometric analysis, techniques such as bibliometric performance analysis, science mapping, and clustering have been employed [32,42].

Regarding bibliometric performance analysis, the following steps were taken: (a) calculation of the articles included per year and per type of review; (b) calculation of the sources and authors with the most documents.

For science mapping, the four techniques presented in Table 2 were used: (a) co-authorship analysis, using authors as the unit of analysis, exploring the collaborative relationships between authors by examining the documents they have co-written; (b) co-occurrence analysis, with author keywords as the unit of analysis, investigating how often specific keywords appear together in the same documents; (c) bibliographic coupling, using sources as the unit of analysis, assessing the degree to which multiple sources reference the same documents; and (d) co-citation analysis, with authors as the unit of analysis, evaluating how frequently two or more authors are cited together in other documents [31,32]. To address potential inconsistencies in journal names, author names, and keywords, thesaurus files were created to correct such discrepancies. For example: injury–injuries, Costa, JA–Costa, J, Applied Sciences (Switzerland)–Applied Sciences. However, no merging was performed for broader synonyms such as soccer–football.

For each technique in Table 2, the following additional elements are provided: (a) the limitations (minimum thresholds) applied to each analysis; (b) the weights, i.e., what determines the size of the node (e.g., number of documents, citations, and occurrences); and (c) the type of visualization (overlay or network). Minimum thresholds (e.g., number of documents, citations, or occurrences) were applied to ensure that the resulting networks remained interpretable and focused on the most influential elements in the field. This is a common practice in bibliometric studies using VOSviewer and is supported by the previous literature [37,43]. In overlay visualization, the color of the nodes is determined by the average publication year of the unit. In contrast, in network visualization, the color of the nodes is determined by the cluster to which each node belongs. Clustering is automatically performed by the software based on the distance on the map and the number of connections [44,45].

## 3. Results

### 3.1. Included Documents

**Figure 1:**
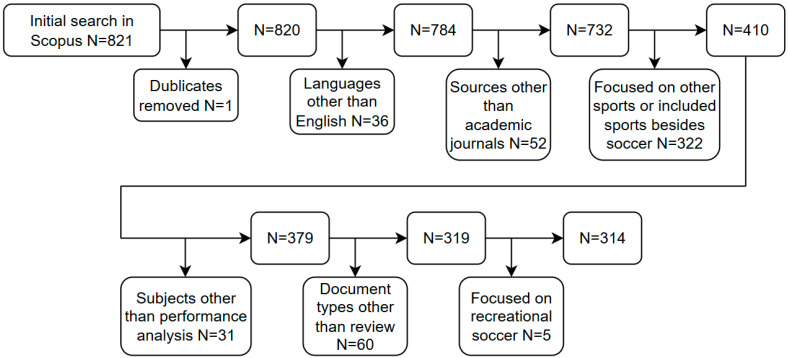
Flow diagram of the article selection process.

As shown in the flow diagram in Figure 1, the initial search in Scopus yielded 821 documents, and 314 documents were ultimately included in our study. The remaining 507 articles were excluded for one of the following reasons: duplicate entry (N = 1), articles written in a language other than English (N = 36), sources other than academic journals (N = 52), articles focused on other sports or included other sports besides soccer (N = 322), primary subject of the article not related to performance analysis (N = 31), document type other than review (N = 60), and articles related to recreational soccer (N = 5).

### 3.2. Bibliometric Performance Analysis

**Figure 2:**
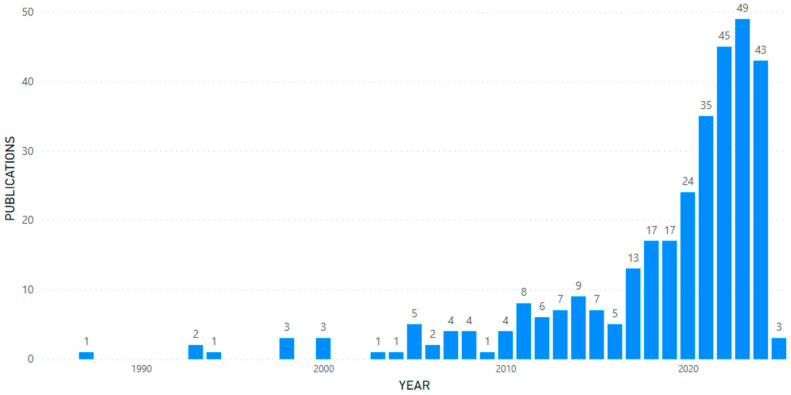
Annual distribution of publications.

The number of publications related to soccer PA has shown a significant increase over the past three decades, as illustrated in Figure 2. From the early 1990s to 2010, the number of publications per year remained relatively low (a maximum of five). From 2011 to 2016, there was a slight increase, with a consistent annual range of five to nine publications. However, from 2017 onwards, a notable upward trend was observed, with consistently over 40 articles per year during the last three years (2022–2024).

**Figure 3:**
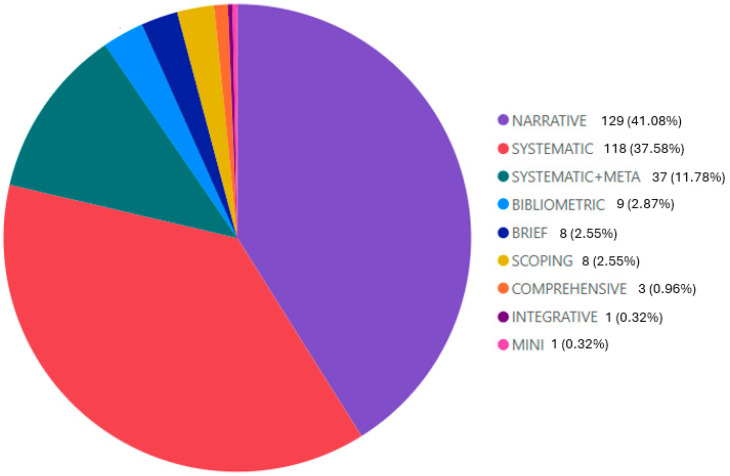
Distribution of review article types in soccer PA research.

The distribution of review article types in soccer PA is presented in Figure 3. The most common type of review article was narrative reviews, accounting for 41.08% (N = 129) of the total publications. Systematic reviews closely followed, with 37.58% of the studies (N = 118). Separately, systematic reviews including meta-analyses accounted for 11.78% (N = 37). Other types of reviews include bibliometric analyses (2.87%, N = 9), brief reviews and scoping reviews (2.55%, N = 8), and comprehensive, integrative, and mini reviews, which collectively constitute a small portion of the research landscape. Interestingly, no umbrella reviews were found among the articles that met the inclusion criteria for our study.

**Figure 4:**
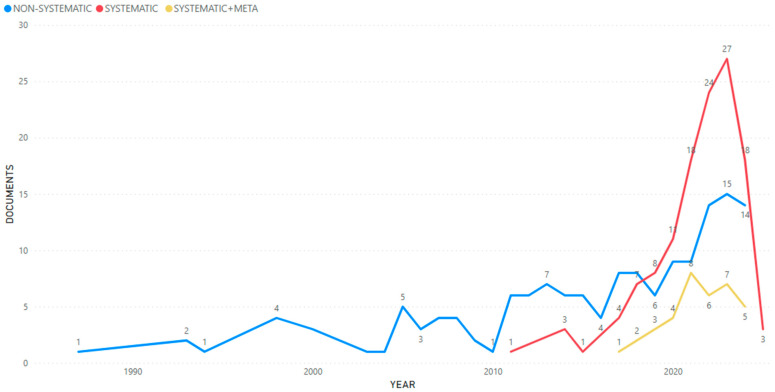
Annual publication trends by type of review (non-systematic, systematic, and meta-analysis).

To examine the evolution of the major types of review articles over time, the different types were grouped into three categories. The first category included articles that used systematic methods (systematic and scoping reviews) without conducting a meta-analysis; the second category focused on meta-analyses; and the third category encompassed all other types (non-systematic). As shown in Figure 4, reviews using systematic methods first appeared in the field in 2011, whereas meta-analyses emerged only in 2017. However, since 2019, systematic review methods have consistently outnumbered non-systematic reviews, with the largest gap observed by 2023.

Table 3 presents the academic sources with the most review articles in the field of soccer PA (the table only shows sources with more than five documents). The sources were ranked according to the number of published documents and total citations. *Sports Medicine* has 36 documents and 7104 citations, highlighting its significant impact on the academic community. *International Journal of Sports Science and Coaching* (17 documents, 472 citations) and *Journal of Sports Sciences* (15 documents, 3970 citations) also demonstrate strong academic contributions, with the latter showing a high citation rate per document. Other notable sources include *Strength and Conditioning Journal*, *Biology of Sport*, and *Sports Medicine—Open*, each contributing substantially to the literature.

Table 4 shows the most prolific authors in the field of soccer PA, highlighting those with more than five review articles. At the top of the list is F. M. Clemente, who has co-authored 30 documents with a total of 985 citations, demonstrating his consistent contribution and influence within the academic community. H. Sarmento ranks second, with 19 articles and 1264 citations, reflecting a high citation impact. R. Ramirez-Campillo and José Afonso also featured prominently, contributing 14 and 13 review articles, respectively. In terms of citation impact, B. Drust stands out with 882 citations from just 8 publications, suggesting that his work holds significant academic influence. Similarly, P. Krustrup achieved an impressive 661 citations with only six documents, underscoring the high quality and impact of his research contributions. The list also includes emerging voices, such as S. Plakias, R. Aquino, and M. Rico-González, who are building a strong presence in the field.

### 3.3. Science Mapping

**Figure 5:**
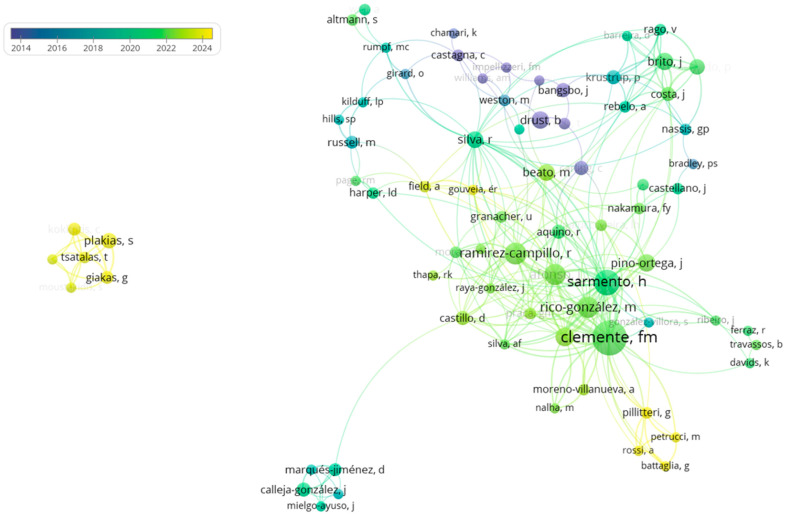
Co-authorship analysis using authors as the unit of analysis.

The co-authorship analysis presented in Figure 5 illustrates the collaborative relationships among authors. Each node represents an author, whereas the links between nodes indicate co-authorship connections. The size of the nodes reflects the number of published documents, and the color of the nodes corresponds to the average publication year, with yellow shading indicating more recent contributions. The largest and most interconnected cluster is centered around F. M. Clemente, demonstrating his extensive collaboration with other leading authors, including Hugo Sarmento, R. Ramirez-Campillo, and M. Rico-González. This cluster indicates a strong and active research network that significantly contributes to the development of knowledge in the field. An interesting observation is the distinct cluster on the left, which includes S. Plakias, T. Tsatalas, and G. Giakas. This cluster appears isolated from the broader network, suggesting that these authors collaborate closely within their research group, but have limited connections with the wider academic community. However, this may also be due to the fact that they are still new to this field, as indicated by the yellow color.

**Figure 6:**
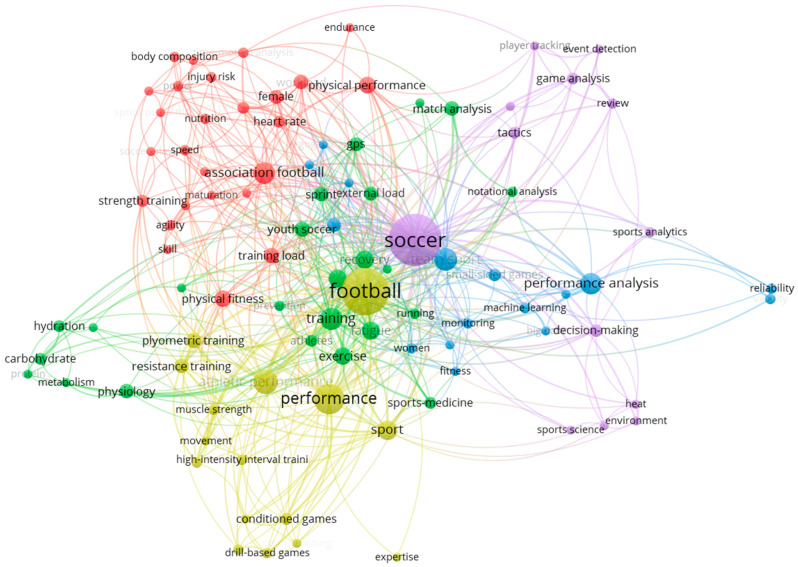
Co-occurrence analysis using author keywords as the unit of analysis. Each color represents a distinct cluster of keywords that co-occur frequently, revealing thematic groupings. The five clusters and their thematic focuses are described in detail in the manuscript text.

The co-occurrence analysis, with author keywords as the unit of analysis, presented in Figure 6, revealed that the keywords were divided into five clusters. The red cluster focuses on training methods and physical parameters related to injury prevention and fitness enhancement, with key terms such as body composition, injury risk, strength training, endurance, physical performance, agility, skill, and speed. The green cluster primarily concerns athlete nutrition, metabolic processes, and recovery techniques, featuring keywords such as hydration, carbohydrate, metabolism, protein, physiology, and sports medicine. The yellow cluster emphasizes coaching strategies, skill development, and high-intensity training methods, with characteristic keywords including training, exercise, conditioned games, drill-based games, high-intensity interval training, muscle strength, and movement. The purple cluster relates to strategic game analysis, the use of technological tools for player monitoring, and decision-making in sports, with keywords such as game analysis, tactics, event detection, player tracking, sports analytics, and decision-making. Finally, the blue cluster appears to focus more on the new era of PA, utilizing big data and machine learning while considering the reliability and validity of the data. Table 5 presents some examples of characteristic articles for each cluster.

**Figure 7:**
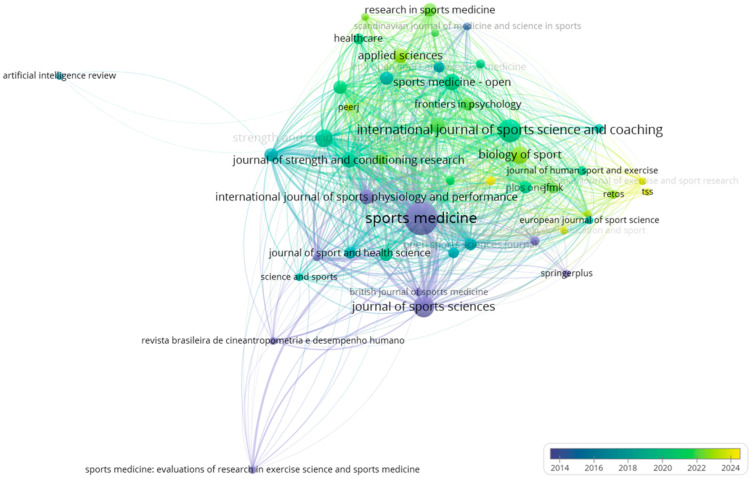
Bibliographic coupling, using sources as the unit of analysis.

The bibliographic coupling analysis presented in Figure 7 assesses the extent to which multiple academic sources reference the same documents. Each node represents a journal, whereas the links between nodes indicate the degree of shared references, with thicker lines suggesting stronger connections. The size of each node reflects the number of documents published by each source, whereas the color gradient (from blue to yellow) represents the average publication year, with yellow indicating more recent publications. The *Sports Medicine* journal is the central and most influential source, demonstrating strong connections with a wide range of other journals, including the *Journal of Sports Sciences*, *International Journal of Sports Science and Coaching*, and *Biology of Sport*. The color of the nodes (yellow, yellow-green) indicates that there are journals that have recently entered the field, but have already made a notable contribution (*Applied Sciences, Journal of Functional Morphology and Kinesiology-JFMK, Trends in Sports Science-TSS*). Another notable observation is the isolated position of the “Artificial Intelligence Review”, which indicates limited integration with the main soccer PA literature. This could reflect a new and developing area for AI applications in sports PA, which has not yet been fully integrated with traditional research.

**Figure 8:**
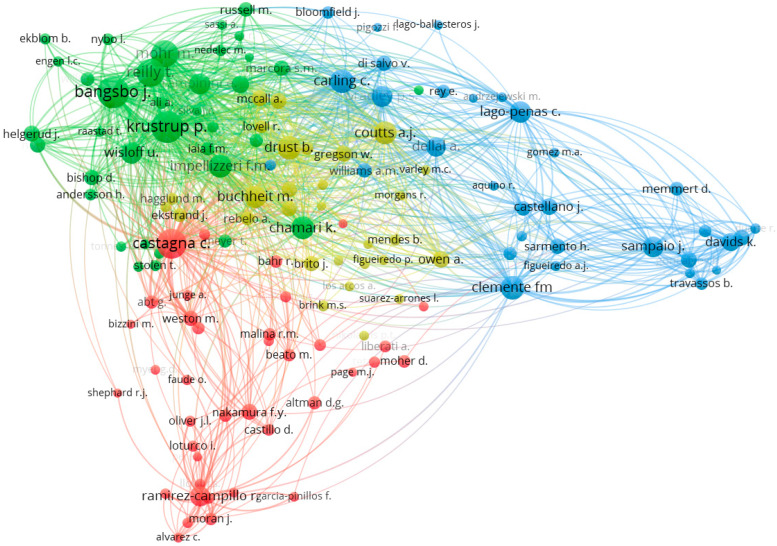
Co-citation analysis using authors as the unit of analysis.

The co-citation analysis presented in Figure 8 evaluates how frequently two or more authors are cited together in other academic documents. The size of each node reflects the total number of citations, while color-coding highlights clusters of closely related authors based on their co-citation patterns. The blue cluster, featuring authors such as F. M. Clemente, H. Sarmento, and C. Lago-Penas, is highly interconnected, suggesting a strong focus on tactics, particularly with an emphasis on decision-making and ecological approaches in soccer research. The green cluster, which includes P. Bangsbo, P. Krustrup, and M. Reilly, focuses on physiological aspects of performance, such as training load, endurance, and physical conditioning. These authors are frequently cited together, reflecting a strong influence on studies related to physical performance and conditioning strategies. In the red cluster, Rodrigo Ramirez-Campillo, J. Moran and M. Beato are key figures, with research emphasis on strength and conditioning, as well as training methodologies such as plyometric training. Finally, the yellow cluster, led by R. Morgans, A. Owen, and B. Buchheit, bridges physiological and tactical approaches, with co-citations indicating a balanced focus on athletic performance metrics and coaching methodologies.

## 4. Discussion

### 4.1. Research Trends and Methodological Issues

As shown in the annual distribution of publications (Figure 2), in recent years (particularly since 2017), there has been an exponential increase in the number of review articles on PA in soccer. The general increase in scientific publications across all fields [68], as well as advancements in sports science and the increasing use of technology in performance monitoring and analysis [62,69,70], provide clear explanations for this phenomenon. However, when we specifically examined review articles on PA in soccer, we observed that non-systematic reviews have remained relatively stable from 2011 to 2021 (Figure 3). 2011 marks the first appearance of systematic reviews, which began to rise sharply from 2018 onwards. Using standardized search protocols, inclusion and exclusion criteria, and study quality assessment, systematic reviews reduce the risk of bias [71,72]. They also offer repeatability [73,74], which is one reason why many journals now accept only systematic reviews and reject other types of review articles [75,76]. Around the same time (2017) as the sudden increase in systematic review publications, we also saw the emergence of systematic reviews combined with meta-analyses. Meta-analyses have the unique ability to objectively summarize existing knowledge from previously published studies that have examined the same topic by using statistical analyses [77]. This approach multiplies the sample size of individual studies, thus increasing the potential for generalizing results and establishing evidence-based guidelines [78]. Overall, the shift towards systematic approaches in review articles reflects a broader trend in scientific research, promoting objectivity and reproducibility in academic publications.

However, the contribution of non-systematic reviews should not be overlooked. Through the qualitative synthesis of findings, many narrative reviews have provided valuable practical advice to practitioners [53,79,80] or have developed important frameworks [65,81,82], offering condensed knowledge (e.g., in a visual format) for practitioners who may not be highly familiar with academic language. Additionally, some authors have used qualitative research methods, such as Grounded Theory [83], to review the literature using the content of previously published articles as data, clarifying concepts, and identifying significant interactions between them. Furthermore, bibliometric analyses can provide useful information on research trends in broad fields, which is not feasible for systematic reviews [31]. Finally, certain opinion papers or critical reviews enhance opportunities for interaction and dialogue among scientists [16,17], which can lead to progress in the field [84]. Therefore, journal editors should encourage such articles. Additionally, an opinion paper may introduce a proposal that could prove important for the field. For example, the suggestion made by Plakias, et al. [85] regarding the integration of qualitative tactical analysis—although commonly used in practical settings by soccer teams—is still not widely recognized at the research/academic level. Naturally, these reviews should result in innovative proposals, conclusions, practical guidelines, and frameworks to make their existence meaningful. It is now quite easy to generate a review, especially with AI applications [86,87]. The real challenge, however, is for authors to use their critical thinking, which AI cannot do, to create innovative proposals and meaningful conclusions.

Moreover, whether a review article is systematic should offer something new and not merely repeat the existing knowledge. Does it create a new framework for the field through qualitative synthesis of the existing literature, or does it simply present known information in chronological or other order? Has it identified gaps in the literature and offered suggestions for future research? Does this highlight the importance of this review and how it might influence the field? Why should a systematic review be published if it lacks a clear research question, and does not truly contribute anything new? What is the value of presenting a methodology regarding the search and selection process for included or excluded articles if, instead of a qualitative synthesis of the findings, it merely lists existing knowledge with lengthy and tiresome tables for the reader?

Finally, meta-analysis is undoubtedly a crucial tool in scientific research, as it provides a more robust and objective assessment of data by combining findings from multiple studies simultaneously [77,78]. Additionally, publication bias can threaten the validity of research findings, especially when only positive studies are published on a specific topic. This is a fairly common phenomenon known as the file-drawer problem, since journals often prefer studies that report statistically significant results [11,88]. Meta-analyses can detect and correct for this bias using techniques such as funnel plots, Egger’s test, or the Trim and Fill method, thus helping to prevent the scientific community from drawing misleading conclusions [89,90]. However, the following should not be overlooked: (a) If a meta-analysis includes low-quality studies, its results will not be reliable (garbage in, garbage out) [91,92]. (b) Sometimes, meta-analyses combine data that are not directly comparable, leading to incorrect or unrealistic conclusions (apples and oranges problem) [93]. (c) There should be a check for homogeneity–heterogeneity of the included studies and the appropriate statistical analysis (fixed-effect or random-effect model, respectively) should be used [94]. A non-exhaustive assessment of the 37 meta-analyses included in this bibliometric analysis revealed the following: (a) Combination of studies examining different training protocols, presenting an apples and oranges problem. (b) Use of a specific model (random or fixed) without justification, i.e., without prior assessment of the homogeneity or heterogeneity of the studies (applying random-effects methods to fixed-effects data does not pose a major problem, but the opposite can have dramatic consequences [11]). (c) Lack of control for publication bias. (d) Use of Egger’s test in meta-analyses that included few studies and high heterogeneity of data [89,95]. (e) Drawing conclusions about publication bias solely from the qualitative assessment of the funnel plot (which inherently involves a degree of subjectivity) while ignoring Egger’s test or the Trim and Fill method, even though these could be used [90].

### 4.2. Leading Authors and Journals

F. M. Clemente and H. Sarmento appear to be leading figures in writing review articles on soccer PA, not only because of the significant number of articles they have contributed (Table 4) but also because of their central position in the co-authorship analysis and co-citation analysis networks (Figure 5 and Figure 8, respectively). These networks not only identified leading authors in soccer PA but also revealed thematic clusters, showing how authors from different research domains are interconnected through shared citations in the academic literature. Specifically, there are researchers who focus on tactics (e.g., S. Plakias, D. Memmert), strength and conditioning (e.g., R. Ramirez-Campillo, M. Beato), physiology (e.g., P. Krustrup, Y. Bangsbo), sports medicine (e.g., G. Nassis, J. Brito), and nutrition (e.g., D. Coutinho, V.H. Teixeira), among others. Naturally, there are researchers who are multifaceted and combine multiple fields. A notable example is C. Carling, who integrates physical performance, tactical analysis, load management, and injury prevention, always with a focus on technology and data analysis to enhance soccer player performance. The collaboration of scientists from different sub-fields, as seen in the co-authorship analysis network (Figure 5), is crucial for progress, as multidisciplinary and interdisciplinary practices are more effective than monodisciplinary approaches [96].

Undoubtedly, the most influential journal in the field is *Sports Medicine*, as it not only ranks first in related publications (Table 3) but also holds a central position in the bibliographic coupling analysis (Figure 7). Given the focus of this bibliometric analysis on soccer PA, the leading position of *Sports Medicine*, a journal primarily dedicated to sports medicine rather than PA specifically within soccer, demonstrates that a significant portion of PA research is centered on the medical–physiological context. Furthermore, the diversity of journals in the network indicates that soccer PA is a multifaceted field, drawing on research from a wide array of disciplines. The presence of specialized sports science journals, such as the *International Journal of Sports Science and Coaching*, the *Journal of Sports Sciences*, and the *International Journal of Performance Analysis in Sport*, alongside multidisciplinary journals such as *Applied Sciences*, highlights both the breadth of influence and interdisciplinary nature of the field. Regarding the peripheral position and limited connections of journals related to AI within the overall network, this is most likely due to the relatively recent emergence of AI-related applications in sports science, as well as possibly limited collaboration between domain experts in soccer and data scientists or AI specialists. To foster integration, we suggest encouraging interdisciplinary collaborations, promoting joint research projects and special issues across domains, and supporting knowledge translation efforts that bridge technological innovation with applied sports contexts. Overall, these sources reflect the diversity of research approaches in soccer PA, ranging from physiological and medical perspectives to coaching and tactical analysis. However, the absence of a journal specifically focused on soccer and the limited number of journals dedicated to sports PA may provide valuable insights to publishers. This could provide an opportunity for the creation and promotion of journals with a dedicated focus on these specific areas, potentially enhancing the visibility and impact of research within the soccer PA community.

### 4.3. Dominant Topics, Research Gaps, and Conceptual Challenges

As discussed regarding the leading authors and journals, soccer PA encompasses numerous subfields, which is also evidenced by the co-occurrence analysis of author keywords (Figure 6). This network not only highlights the thematic diversity of the field but also shows the potential for collaboration across different scientific domains. The division of keywords into five distinct clusters illustrates the complexity of PA in soccer, in which medical, physiological, coaching, tactical, and technological topics coexist. For example, while the red cluster focuses on physical parameters and injury prevention, the purple cluster emphasizes the use of modern technologies and analytics to enhance tactical analysis and decision-making. This contrast underscores the field’s shift from traditional physiological approaches to data-driven methods, leveraging big data and machine learning (blue cluster). Furthermore, the coexistence of the green and yellow clusters demonstrates the close connection between nutrition and physiology with coaching strategies, indicating that athletic performance depends not only on physical capacity, but also on factors such as nutrition, recovery, and proper preparation. This holistic approach could serve as a key point for improving soccer PA practices, as it underscores the importance of integrating diverse scientific knowledge into the training environment.

Despite the broad spectrum of topics covered by review articles on soccer PA, this study identified critical gaps in the existing literature. Notably, there is a distinct lack of review articles focusing on set pieces (e.g., corners, free kicks, penalties, throw-ins), key performance indicators (KPIs), and contextual variables. Set pieces are pivotal in soccer and often serve as decisive moments that influence the outcome of matches [97,98]. KPIs that objectively evaluate and guide performance events are associated with team success [99]. As a consequence of the lack of review articles focusing on KPIs, there is also a noticeable scarcity of studies addressing topics such as predicting match outcomes and identifying the winning formula. Moreover, considering contextual variables is now recognized as a fundamental aspect of PA, ensuring that evaluations are not only accurate, but also relevant to the specific circumstances of each match. Without this context, analyses risk leading to misleading or oversimplified conclusions [100]. Finally, it is worth noting that only two review articles specifically address the defensive phase [27,62], as most authors appear to place greater emphasis on the ball possession phase. The absence of review articles on these crucial topics is surprising and concerning, highlighting a clear opportunity for future research. Addressing these gaps could provide valuable insights and practical guidance to coaches, analysts, and researchers, ultimately contributing to more effective performance strategies and a deeper understanding of the factors that influence soccer success. Therefore, future scholars should prioritize these underexplored areas to meaningfully advance the field of PA.

Beyond the dominant topics and identified research gaps, two additional issues of considerable importance have been highlighted by various authors. The first concerns the persistent gap between researchers and practitioners, with many studies emphasizing the need to bridge this divide [13,85,101,102,103]. The second pertains to the lack of consensus regarding the functional definitions employed across different documents. This inconsistency in terminology and conceptual frameworks hinders the comparability of findings and the development of cumulative knowledge [30,81,104]. Addressing these two challenges, bridging the research–practice divide and promoting terminological clarity, should be considered essential priorities for future research in the field of soccer performance analysis.

### 4.4. Some Practical Considerations

Based on the existing literature of review articles on soccer PA, we offer the following suggestions for future authors, reviewers, and publishers:

- Authors should include the term “soccer” in the title, abstract, or keywords, allowing readers to easily and quickly determine whether the article pertains to soccer specifically or to another type of football, such as rugby. Particularly unhelpful were instances where the term “football” appeared in both the title and keywords, without any mention of “soccer”.
- The methodology followed and the type of review conducted should be specified clearly. Some review articles did not provide any label or type of review, while others used labels that did not accurately reflect their methodology. For example, we encountered articles labeled as “brief reviews”, with some following a narrative methodology and others using systematic methods, or as “comprehensive reviews”, without clarity regarding their methodological approach.
- Journals should not avoid publishing non-systematic reviews, provided that they truly offer something new, present a clear research question, and lead to a conclusion or framework that can assist future authors or practitioners. A review article, whether systematic or narrative, should contribute to the field by offering fresh insights, identifying research gaps, and proposing innovative frameworks. Its purpose should not be to simply repeat what is already known, but to challenge the status quo and open the way for future research and practical applications.
- Regardless of the review type, authors should strive to connect their findings to real-world applications. Practical recommendations for coaches, analysts, and practitioners can enhance the impact of review articles and bridge the gap between research and practice.
- While meta-analyses offer significant advantages in synthesizing evidence, their validity and reliability depend heavily on methodological rigor. If meta-analyses contain errors, such as those mentioned earlier in the last paragraph of Section 4.1, the conclusions drawn may range from incomplete to misleading.

Journals could implement guidelines for reviewers of review articles, ensuring that they assess not only the methodological rigor, but also the originality, practical value, and clarity of the review’s contribution to the field. However, journals may face several practical challenges in implementing these suggestions. These include limited editorial time and staff, difficulty in distinguishing between high- and low-quality non-systematic reviews, and the absence of standardized guidelines for narrative or integrative reviews. In addition, some journals may be hesitant to change established practices that favor systematic reviews, while providing proper guidance to reviewers for evaluating diverse types of reviews can also be demanding. Addressing these barriers would likely require coordinated efforts across editorial boards and the broader academic community.

### 4.5. Limitations

This study had several limitations that should be acknowledged to provide a balanced interpretation of the findings. First, the analysis was based solely on data retrieved from the Scopus database, which, despite its comprehensive coverage, may not include all relevant review articles on soccer PA. Articles indexed in other databases, such as SPORTDiscus, Web of Science, PubMed, and Google Scholar, might have been overlooked, potentially affecting the completeness of the dataset. Nevertheless, the aim of the bibliometric analysis is not to provide a comprehensive review, but rather to identify and illustrate prevailing trends in the international literature—something that can only be effectively accomplished using records indexed in Scopus [105]. Second, this study focused exclusively on English-language publications, which may have introduced a language bias and excluded relevant research published in other languages. Considering the global popularity of soccer, this limitation may have led to the exclusion of valuable insights from non-English-speaking countries. For example, omitting non-English review articles may lead to the underrepresentation of region-specific themes, such as culturally embedded coaching strategies, locally relevant performance indicators, or context-specific methodological approaches, which could enrich the global understanding of soccer PA. Additionally, this study primarily assessed published review articles, excluding grey literature such as conference proceedings, preprints, theses, and books. These sources might contain emerging research trends or practical insights, particularly in a rapidly evolving field such as soccer PA, where new technologies and innovative methodologies are constantly being developed. Finally, the bibliometric analysis conducted with VOSviewer relied on author-provided keywords and metadata. The variability in keyword selection and inconsistency in terminology used by different authors may have impacted clustering and thematic analysis, possibly resulting in thematic overlaps or missed connections between related topics.

## 5. Conclusions

This bibliometric analysis examined the evolution of review articles on PA in soccer, identifying leading authors, journals, and dominant research topics. The study revealed an exponential increase in review articles since 2017, with a shift towards systematic reviews and meta-analyses. F. M. Clemente and H. Sarmento emerged as the leading authors, while *Sports Medicine* was the most influential journal. The analysis highlights the multidisciplinary nature of soccer PA, encompassing medical, physiological, coaching, tactical, and technological aspects. However, this study also identifies critical research gaps, such as the lack of reviews on set pieces, key performance indicators, and contextual variables. We provide practical suggestions for future research, emphasizing the need for originality, practical value, and methodological rigor in the review articles. Despite limitations, such as the exclusive use of the Scopus database and the focus on English-language publications, this study offers valuable insights into the current state and future direction of writing review articles on soccer PA.

## Figures and Tables

**Table 1:** Inclusion and exclusion criteria for article selection.

Inclusion Criteria	Exclusion Criteria
Articles explicitly categorized as review articles or theoretical articles that synthesize existing literature	Empirical studies (quantitative or qualitative) presenting original data
Written in English	Articles written in languages other than English
Published in peer-reviewed academic journals	Articles published in non-academic sources (e.g., magazines, conference abstracts, reports)
Primary focus on performance analysis (PA) in soccer	Articles that do not focus on soccer or that discuss soccer alongside other sports without clear focus
Concerned with active soccer players of any sex, competitive level, or age	Studies focused exclusively on recreational or non-competitive players
Use of the term “performance analysis” in its broadest sense (technical, tactical, physical, physiological, and psychological aspects)	Articles unrelated to aspects of performance or not contributing to the understanding of soccer performance

**Table 2:** Overview of bibliometric analysis techniques and applied parameters in VOSviewer (version 1.6.20.0, Centre for Science and Technology Studies, Leiden University, Leiden, Netherlands).

Technique	Unit of Analysis	Limitations (Minimum Thresholds)	Weights	Type of Visualization
Co-authorship	Authors	(a) Minimum number of documents of an author = 3. (b) Only authors linked to at least one other author.	Documents	Overlay
Co-occurence	Author keywords	Minimum number of occurrences of a keyword = 3.	Occurences	Network
Bibliographic coupling	Sources	(a) Minimum number of documents of a source = 2. (b) Only items connected to each other.	Documents	Overlay
Co-citation	Authors	Minimum number of citations of an author = 50.	Citations	Network

**Table 3:** Sources of more than five review articles on soccer performance analysis.

Source	Documents	Citations
*Sports Medicine*	36	7104
*International Journal of Sports Science and Coaching*	17	472
*Journal of Sports Sciences*	15	3970
*Strength and Conditioning Journal*	10	323
*Biology of Sport*	10	199
*Sports Medicine—Open*	9	394
*International Journal of Sports Physiology and Performance*	7	838
*Journal of Strength and Conditioning Research*	7	201
*Sports*	7	68
*Applied Sciences*	7	31
*International Journal of Performance Analysis in Sport*	6	336
*Journal of Sports Medicine and Physical Fitness*	6	291
*International Journal of Environmental Research and Public Health*	6	168
*Frontiers in Psychology*	6	164
*Research in Sports Medicine*	6	139

**Table 4:** Leading authors with more than five review articles on soccer PA.

Author	Documents	Citations
Clemente, Filipe Manuel	30	985
Sarmento, Hugo	19	1264
Ramirez-Campillo, Rodrigo	14	327
Afonso, José	13	270
Rico-González, Markel	13	223
Oliveira, Rafael	10	156
Brito, João	9	179
Pino-Ortega, José	9	108
Drust, Barry	8	882
Beato, Marco	8	184
Figueiredo, Pedro	7	160
Plakias, Spyridon	7	55
Krustrup, Peter	6	661
Aquino, Rodrigo	6	251
Castillo, Daniel	6	210
Calleja-González, Julio	6	112

**Table 5:** Examples of articles for each cluster.

Cluster	Article Citations
Red	[46,47,48,49,50]
Green	[51,52,53,54,55]
Yellow	[56,57,58,59,60]
Purple	[13,28,61,62,63]
Blue	[24,64,65,66,67]

## Data Availability

The data are available at https://zenodo.org/records/14917486, accessed on 24 February 2025.
